# Transcriptome and Proteome Profiling of Neural Induced Pluripotent Stem Cells from Individuals with Down Syndrome Disclose Dynamic Dysregulations of Key Pathways and Cellular Functions

**DOI:** 10.1007/s12035-019-1585-3

**Published:** 2019-04-13

**Authors:** Maria Sobol, Joakim Klar, Loora Laan, Mansoureh Shahsavani, Jens Schuster, Göran Annerén, Anne Konzer, Jia Mi, Jonas Bergquist, Jessica Nordlund, Jan Hoeber, Mikael Huss, Anna Falk, Niklas Dahl

**Affiliations:** 1 Department of Immunology, Genetics and Pathology, Science for Life Laboratory, Uppsala University, Box 815, SE-751 08 Uppsala, Sweden; 2 Department of Neuroscience, Karolinska Institutet Solna, SE-171 65 Stockholm, Sweden; 3 Department of Chemistry – BMC, Analytical Chemistry, Uppsala University, Box 599, SE-751 24 Uppsala, Sweden; 4 Department of Medical Sciences and Science for Life Laboratory, Uppsala University, Box 1432, SE-751 44 Uppsala, Sweden; 5 Department of Biochemistry and Biophysics, National Bioinformatics Infrastructure Sweden, Science for Life Laboratory, Stockholm University, Box 1031, SE-171 21 Solna, Sweden

**Keywords:** Down syndrome, Induced pluripotent stem cells (iPSC), Neural differentiation, RNA sequencing, Proteome profiling

## Abstract

Down syndrome (DS) or trisomy 21 (T21) is a leading genetic cause of intellectual disability. To gain insights into dynamics of molecular perturbations during neurogenesis in DS, we established a model using induced pluripotent stem cells (iPSC) with transcriptome profiles comparable to that of normal fetal brain development. When applied on iPSCs with T21, transcriptome and proteome signatures at two stages of differentiation revealed strong temporal dynamics of dysregulated genes, proteins and pathways belonging to 11 major functional clusters. DNA replication, synaptic maturation and neuroactive clusters were disturbed at the early differentiation time point accompanied by a skewed transition from the neural progenitor cell stage and reduced cellular growth. With differentiation, growth factor and extracellular matrix, oxidative phosphorylation and glycolysis emerged as major perturbed clusters. Furthermore, we identified a marked dysregulation of a set of genes encoded by chromosome 21 including an early upregulation of the hub gene *APP*, supporting its role for disturbed neurogenesis, and the transcription factors *OLIG1*, *OLIG2* and *RUNX1*, consistent with deficient myelination and neuronal differentiation. Taken together, our findings highlight novel sequential and differentiation-dependent dynamics of disturbed functions, pathways and elements in T21 neurogenesis, providing further insights into developmental abnormalities of the DS brain.

**Electronic supplementary material:**

The online version of this article (10.1007/s12035-019-1585-3) contains supplementary material, which is available to authorized users.

## Introduction

Down syndrome (DS) is the most common specific cause of intellectual disability with an incidence of approximately 1/750 births and without ethnical predilections [1]. Most individuals with DS have a complete trisomy for chromosome 21 (HSA21) leading to a complex phenotype. Impaired cognition is a major disabling feature in DS, and this is associated with gross regional and cellular brain abnormalities [2–4]. Studies of post-mortem human brain specimens with T21 and orthologous mice models have revealed a range of disturbed processes in DS neural cells such as proliferative rate, oxidative stress, glia cell fates, myelination, neurite outgrowth and synaptic formation [4–9]. It is now generally accepted that DS is the result of complex and global transcriptomic changes induced by genomic imbalance of HSA21 and HAS21 encoded transcription factors [9–13].

The limited access to brain specimens and the requirement for high throughput analysis have made neural derivatives from induced pluripotent stem cells (iPSCs) with T21 an attractive in vitro model of DS. Independent studies using iPSC derivatives with T21 have recently recapitulated several DS brain abnormalities with respect to gene expression, neural progenitor cell proliferation [14–16], neurogenesis [17, 18], glia cell fate and glia cell function [19], neurite outgrowth [20], synaptic morphology and mitochondrial function [16, 21] and amyloid deposition with an Alzheimer like pathology of cortical neurons [22]. While the analysis on molecular profiles in brain specimens and iPSC-derived neural cells with T21 has been crucial for insights into mechanisms behind abnormal neurogenesis in DS, the studies have been limited by the differentiation protocols used to generate neuronal cells, by the developmental time points analysed or by the sensitivity and methodologies used for molecular profiling. Three studies have undertaken deep RNA sequencing (RNA-seq) on iPSC with T21 [12, 15, 18], but the assessments were not performed on differentiated neural cells and did not uncover temporal dynamic abnormalities along neurogenesis.

Because the anatomical and cellular brain abnormalities in DS are established at birth, the therapeutic strategies to improve cognition should ideally be tailored to interfere with neurodevelopmental abnormalities. Therefore, identification of druggable pathways and elements along differentiation of trisomic neuronal cells is important [12, 13, 23–25]. To this end, we established iPSC-derived neural cell model showing transcriptomic similarities to that of two distinct differentiation stages of the fetal brain. Using this model, we assessed the protein coding transcriptome and the proteome associated with T21. Integrated analysis of our data sets revealed dynamic perturbations of major functional clusters, hub proteins and HSA21 genes that are accompanied by cellular abnormalities. The combined data generated from our model shed further light on neurogenesis in T21 cells with implications for DS brain development.

## Materials and Methods

### Generation and Maintenance of iPSCs

We established iPSC with HSA21 by transducing fibroblast cells from one male and one female (DS1 and DS2) with characteristic DS features and a full T21 with Sendai virus mediated transgenic transduction of the four factors OCT4, SOX2, KLF4 and c-MYC (CytoTune®-iPS Sendai Reprogramming Kit, Gibco). Three iPSC lines from age-matched healthy donors (Ctrl1, Ctrl2 and Ctrl9) were previously established and characterized for pluripotency [26–28]. Transduced cells (passage (P) 1–3) were plated in 24-well plates (Corning) with pre-warmed HFF medium (DMEM (Sigma), 15% FBS (Sigma), 2% penicillin–streptomycin (Gibco), 1% non-essential amino acids (Gibco) and cultured under standard conditions for 6 days. Day 7 after transduction, we seeded 1.5 × 10^5^ cells from every cell line onto 100-mm dishes pre-plated with 3.5 × 10^6^ mitomycin C-inactivated HFFC feeder cells in HFF medium. Medium was then replaced every day with human embryonic stem cells (hESCs) medium (Knockout DMEM, Knockout Serum Replacement, non-essential-amino acids, Glutamax, penicillin–streptomycin (Gibco) and 10 ng/mL recombinant human (rh) FGF-basic (R&D systems)) until picking of P0 colonies at days 21–28 after transduction. The iPSCs were picked clonally and separate clones were cultured on feeder cells. Overnight treatment with 5 μM ROCK inhibitor (Y-27632, Cellagentech) was used to enhance cell survival. Standard karyotype analysis confirmed T21 in iPSC derived from both DS patients, respectively (Supplementary Fig. 1a). The iPSC lines expressed pluripotent markers OCT4, SSEA4, NANOG and TRA-81, and transcriptome data from our iPSCs were further validated using the PluriTest web-portal (www.pluritest.org; [29]; Supplementary Fig. 1b) online for comparison with approximately 450 transcriptional profiles from 223 hESCs and 41 human iPSC lines [29].

The sample donors for this study or their parents/legal guardians have signed written informed consents to provide samples for generation of T21 iPSCs and further differentiation. The experimental protocol was reviewed and approved by the regional ethical committee of Uppsala, Sweden (Dnr 2016/209).

### Neural Induction and Differentiation

We first differentiated selected lines into neural progenitor cells (NPCs) representing a stable neural progenitor stage expressing the markers PAX6 and NESTIN [30]. iPSC lines were cultured on feeder cells until about 20% confluence and treated with 2 μM DAPT (Sigma), 10 ng/mL hLIF (Millipore), 3 μM CHIR99021 (Cellagentech) and 2 μM SB431542 (Cellagentech) in neural induction media containing DMEM/F12:Neurobasal (1:1), 1× N2, 1× B2 and 1% Glutmax (all from Gibco) for 7 days. After 5–7 days, neuronal rosettes were picked and placed to low-attachment plate in DMEM/F12 medium with N2 supplement (1:100, Gibco) for 2 days. Floating rosettes were dissociated in TrypLE-Express (Gibco) to single cells and plated onto 0.1 mg poly-l-ornithine (Sigma) and 1 μg/mL laminin (L2020, Sigma)-coated plates at density 40 × 10^3^ cells per 1 cm^2^ in medium containing DMEM/F12 GlutaMAX (Gibco) supplemented with 10 ng/mL rhFGF-basic (R&D systems), 10 ng/mL recombinant human epidermal growth factor (rhEGF) (R&D systems), B27 supplement (1:1000, Gibco), N2 supplement (1:100, Gibco) and 1% of penicillin/streptomycin (Gibco). Established NPC lines were passaged at the ratio of 1:3 every second to third day using TrypLE Express (Gibco) and defined trypsin inhibitor (Gibco) as described [30].

Non-directed neuronal differentiation was induced in NPCs by removal of rhFGF-basic and rhEGF. Fully confluent NPCs were passaged on poly-l-ornithine–laminin–coated plates with average density 25 × 10^3^ cells per cm^2^ in the NPC medium described above. Next day the medium was changed to a “differentiation” medium containing DMEM/F12 GlutaMAX supplemented with 1% N2, B27 and penicillin/streptomycin. Cells were cultured in this medium up to 30 days with half media change every second day.

### Karyotyping

Chromosome preparation was performed following protocol described previously [31]. Metaphases were analysed using Metafer slide scanning platform and IKAROS-software (MetaSystems). Twenty metaphases were analysed for each cell line.

### Immunofluorescence

NPCs and neural cells were cultured on poly-l-ornithine–laminin–coated coverslips (initial density 22 × 10^3^ cells per cm^2^) for 2 days and up to 30 days respectively prior to IF labelling. IF staining was performed using standard techniques [32]. Primary antibodies against Nestin (1:100, R&D Systems), Pax6 (1:100, Covance), β-III-tubulin (1:80, Sigma), GFAP (1:500, Sigma), Vimentin (1:500, Abcam) and Caspase-3 (1:400, Cell Signaling) were bind overnight at 4 °C. After washing in 1× TBS, 0.05% Tween, α-mouse IgG AlexaFluor 488 and α-rabbit IgG AlexaFluor 555 (1:10000, Invitrogen) were applied alone or in appropriate combinations for 1.5 h at room temperature in the dark. Visualization was performed on Zeiss 510 confocal microscope (Carl Zeiss microscopy) using Zen 2009 imaging software. Image analyses were performed using ImageJ software.

### Long-Term Live Cell Imaging

To determine the proliferative rate, we plated 5 × 10^4^ cells per well in 24-well plates (Corning) and cultured in standard NPC media. Cells were monitored using the IncuCyte ZOOM™ live cell imaging system (Essen BioScience, MI). Images of proliferating cells were taken every second hour during next 96 h after the cell seeding. Experiments were performed in triplicates for each cell line. Proliferation analysis was performed using the IncuCyte integrated software module for proliferation assay. Visualization for long-term live cell imaging and motility was performed as described above. The cells were captured every 30 min and tracked during 6 h. Manual cell tracking was performed using ImageJ software and TrackMate plugin.

### Neurite Outgrowth

Neurites in NPCs derived from DS1, DS2 Ctrl1 and Ctrl2 were visualised and counted using β-III-tubulin staining as described above. In DS NPCs, 101 cells were analysed and, in Ctrl NPCs, 148 cells were analysed. Statistical analysis was performed using one-way analysis of variance (ANOVA) followed by Tukey’s post hoc test in PractiStat software.

### RNA/DNA Isolation

Total RNA extraction was performed with the PureLink RNA Mini Kit including on column DNAse treatment following the manufacturer’s instructions (Life Technologies). cDNA was synthesised from 1 μg of total RNA using Superscript VILO cDNA synthesis kit (Life Technologies). Genomic DNA was isolated using NucleoSpin Kit following the manufacturer’s instructions (Macherey-Nagel).

### Analysis of ROS

Stress sensitivity of cultured neuronal cells was analyzed using the OxiSelect™ Intracellular ROS Assay Kit (Cell Biolabs) following manufacturer’s protocols. Briefly, NPC derived neuronal cells at day 30 were loaded with 2′,7′-dichlorodihydrofluorescin diacetate (DCFH-DA) for 1 h. Subsequently, production of reactive oxygen species (ROS) was followed by monitoring increase in fluorescence (excitation 480 nm; emission 530 nm) for 120 min using a VarioScan LUX (Thermo Fisher Scientific) plate reader. A second set of cells was treated with hydrogen peroxide to induce cellular stress immediately before fluorescence measurement. Data was normalized to fluorescence at start of the measurement (*t* = 0) and was plotted as relative fluorescence units using Prism showing change of ROS load over time.

### RNA Sequencing, Transcriptome Analysis and Mitochondrial DNA Quantification

Paired-end RNA-sequencing was performed using Illumina HiSeq (Illumina). The sequencing reads were aligned to the ENSEMBL human reference genome (Homo_sapiens.GRCh37.75), and gene counts were generated using the STAR read aligner [33]. To cluster our data with Brainspan, we downloaded the file expression_matrix.csv and associated annotation files from http://brainspan.org and transformed for comparison to our gene counts. Clustering of samples was performed using a gene set generated by selecting the 30 most differentially expressed genes between iPSCs, NPCs and differentiated neural progenitor cells (DiffNPCs) derived from the controls (Ctrl1 and Ctrl2; see Supplementary Table 7). Analysis of the count data to identify differentially expressed (DE) transcripts was performed using the DESeq2 package using a Benjamini and Hochberg adjusted *p* value < 0.05 for cut-off [34]. The functional annotations (KEGG Pathway, GO Molecular Function, Chromosomal Location, PPI Hub Proteins) of DE genes and proteins in T21 cells compared to control were performed using the web-based annotation tool Enrichr (http://amp.pharm.mssm.edu/Enrichr/). The web-based annotation tool Enrichr was used for functional annotations of DE gene and functional annotation of clustering was performed by using the Database for Annotation, Visualization and Integrated Discovery (DAVID) Bioinformatics Resource 6.8 (https://david.ncifcrf.gov) using data from NPC and DiffNPC lines separately. The RNA-sequencing data was validated using StepOnePlus™ Real-Time PCR System (Applied Biosystems) using primers for 10 selected transcripts, and quantification of mitochondrial DNA was determined using ddPCR system including an automated droplet generator and reader (QX200 Droplet Digital PCR, Bio-Rad; [35]; Supplementary Materials and Methods).

### Mass Spectrometry and Proteome Analysis

The sample preparation was performed according to a protocol provided by Dr. Anne Konzer [36]. The peptides were purified and electrosprayed online to a Q Exactive Plus Orbitrap mass spectrometer (Thermo Finnigan). Tandem mass spectrometry was performed applying HCD. Protein identification and quantitation was performed using the quantitation software MaxQuant 1.5.1.2 (Supplementary Materials and Methods). The RAW data files from each comparison were combined into one search respectively in the software. The database for protein identification contains human proteins extracted from the Swissprot database (Release April 2015). Differentially expressed proteins (DEP) were defined using a Bonferroni corrected two-tailed probability of the chi-squared distribution (corrected *p* value < 0.05).

## Results

### Assessment of the iPSC to Model Neurogenesis

**Fig. 1:**
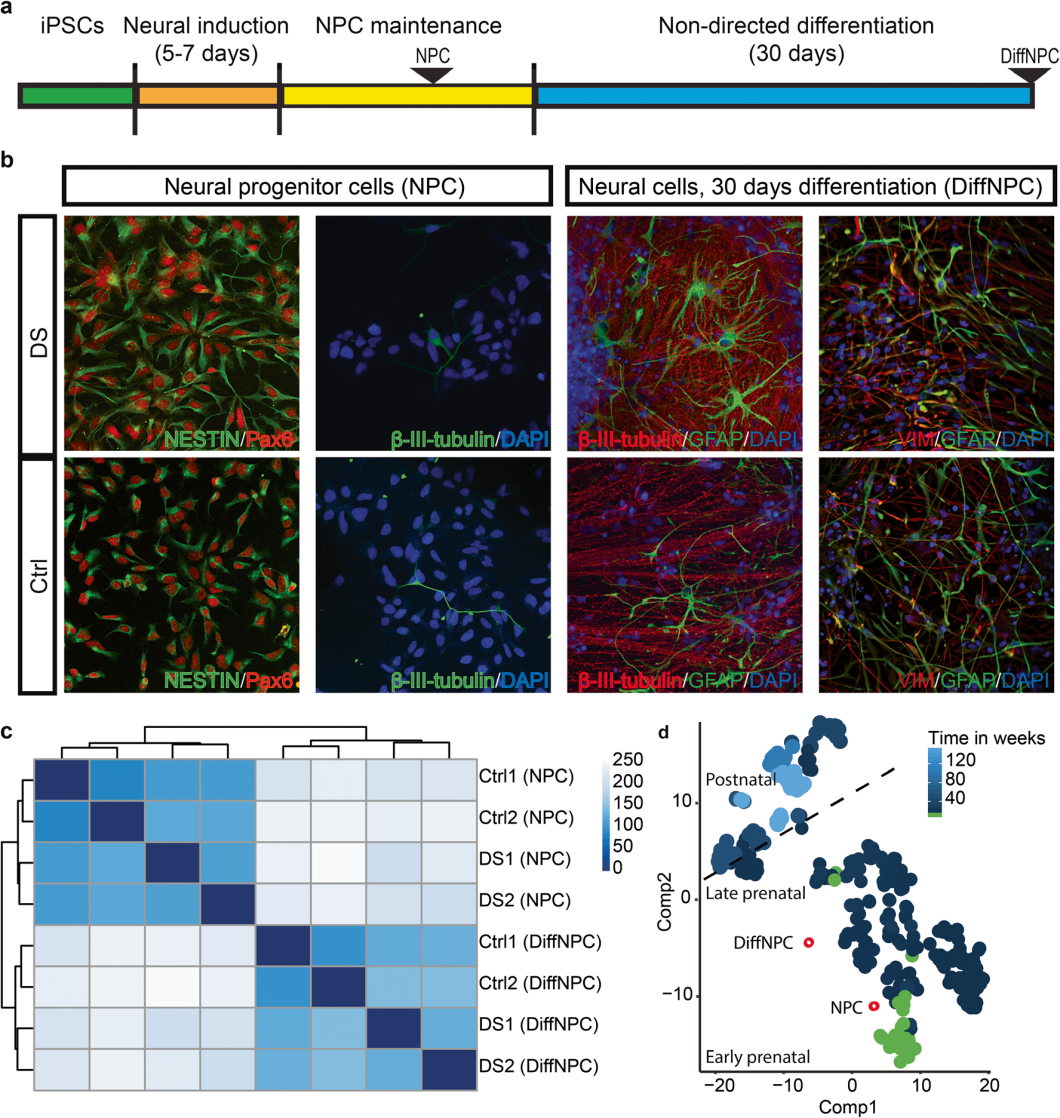
Generation and characterization of the iPSC model. **a** Schematic presentation of the protocol used to generate NPCs and DiffNPCs from iPSCs. **b** Representative images of immunofluorescent stainings of cells with full trisomy 21 (DS) and euploid control (Ctrl) cells. Panels show neural progenitor cells (NPC) stained for NESTIN and Pax6 as well as differentiated neural progenitor cells (DiffNPCs; 30 days of non-directed differentiation) stained for β-III-tubulin/TUJ1, GFAP and Vimentin. **c** Heatmap of transcriptome sample-to-sample distances using the rlog-transformed values (DS1 and DS2: neural iPSC lines with T21; Ctrl1 and Ctrl2: euploid neural iPSC lines). **d** Clustering of transcriptome data from neural iPSC lines derived from the two healthy donors and at two differentiation time points (NPC and DiffNPC; red circles) together with transcriptomes from Brainspan brain samples of different post-conceptional weeks (pcw) using tSNE plot. Brainspan samples represent fetal ages up to 200 pcw. Early fetal samples at age between 8 and 10 pcw are coloured green (*n* = 30) and remaining fetal samples are coloured in different blue shades corresponding to their gestational ages > 10 pcw. The dashed line indicates estimated position of clusters at full term. Clustering of samples was performed using the key gene set

We reprogrammed fibroblasts from two DS patients, one male and one female (DS1 and DS2, respectively), with characteristic DS features and full T21. The iPSCs were induced to a self-renewing neural progenitor cell (NPC) stage with a defined marker profile and to a more differentiated neural stage (DiffNPC) by non-directed differentiation for 30 days [30] together with previously characterised iPSCs derived from three age-matched healthy donors (Ctrl1, Ctrl2, and Ctrl9, respectively; Fig. 1a). The NPC and the DiffNPC differentiation stages were characterized by staining with relevant neuronal markers (Fig. 1b) and by karyotyping. We further obtained genome wide RNAseq data from the four cell lines at both the NPC and DiffNPC stages. The number of reads obtained from RNAseq in each sample was comparable (average 78.9 M, range 60.8–100.2 M paired-end reads/sample). Clustering analysis of the normalized expression data showed that the two T21 lines grouped pairwise at the NPC and DiffNPC stages, respectively, and with a distinct transcriptome profile compared to control cells (Fig. 1c). To address how our cultures related to stages of normal brain development, we obtained gene expression data from the Brainspan samples representing 398 samples (http://www.brainspan.org) and compared them to our RNAseq data. Using t-distributed stochastic neighbour embedding (t-SNE), we observed that our NPCs clustered close to brain transcriptomes corresponding to an early fetal stage (< 13 post-conceptional (p.c.) weeks; Fig. 1d). The RNAseq profiles of DiffNPCs, however, clustered closer to that of the brain at approximately 20–30 p.c. weeks. These data suggest that our cell model exhibit transcriptome profiles with similarities to the developing brain and that the expression profiles of T21 lines cluster together, distinct from that of euploid lines.

### Transcriptome and Proteome Analysis Reveal Extensive Dysregulations in Neural Lines with T21

**Fig. 2:**
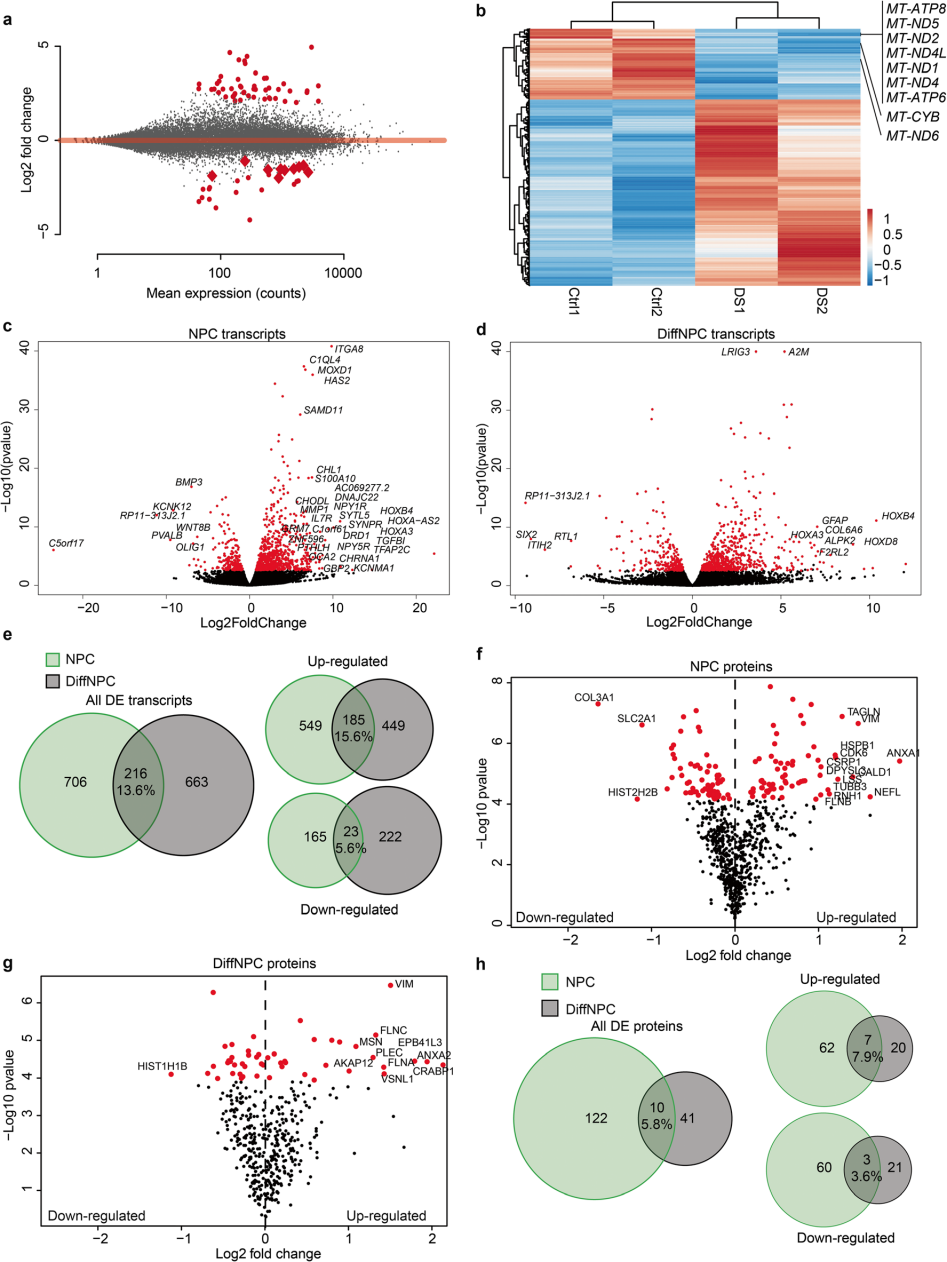
Differential levels of transcripts and proteins in T21 lines. **a** Overview of transcript levels in T21 lines (DS1 and DS2) relative that in euploid control lines (Ctrl1 and Ctrl2) at the DiffNPC stage evaluated using DESeq2. The log2fold change of expression levels in T21 lines is shown vs. the mean expression levels in control lines. Red dots correspond to significant differential expression (DE; adjusted *p* value < 0.05) levels with a log2fold change of > 1. Grey dots correspond to non-significant difference in expression levels. Mitochondrial (MT) transcript levels are annotated with red diamonds, all of them downregulated in T21 lines. **b** Heatmap and clustering of DE transcripts in DiffNPC from control lines (Ctrl1 and Ctrl2) and the two T21 lines (DS1 and DS2). Data from MT transcript levels are annotated as reference. **c** Volcano plots of differentially expressed genes (DEGs; red; adjusted *p* value < 0.05) from comparison of T21 lines (DS1 and DS2) and euploid lines (Ctrl1 and Ctrl2) at the NPC stage. Selected DEGs are named. **d** Volcano plots of DEGs as in **c** but at the DiffNPC stage. **e** Venn diagrams showing all DE genes when comparing T21 and euploid lines (left panel) at the NPC (green) and DiffNPC (grey) stages of differentiation. The overlap in the Venn diagram therefore represents DE genes regardless of differentiation stages. Right panels show DE genes in T21 lines separated by up- and downregulated genes, respectively. **f** Volcano plots of differentially expressed (DE) proteins (red) from pairwise comparisons of T21 lines (DS1 and DS2) and euploid lines (Ctrl1 and Ctrl2) at the NPC stage. Selected DE proteins with log2 fold change < − 1 and > 1 are annotated with names. **g** DE proteins (red) from pairwise comparisons of T21 lines (DS1 and DS2) and euploid lines (Ctrl1 and Ctrl2) at the DiffNPC stage. **h** Venn diagram showing all DE proteins when comparing T21 and euploid lines (left) at the NPC (green) and DiffNPC (grey) stages of differentiation. The overlap in the Venn diagram therefore represents DE proteins regardless of differentiation stage. Right panels show DE proteins in T21 lines separated by up- and downregulated proteins, respectively

We first analysed our transcriptome data for differentially expressed genes (DEGs) in T21 lines. Analysis of fold changes showed a distribution of both up- and downregulated transcripts in trisomic lines (Fig. 2a). Furthermore, the expression levels of individual transcripts showed a strong pairwise clustering of the trisomic vs. euploid lines (Fig. 2b). In trisomic NPCs, we identified 922 DEGs when compared to the euploid lines (adjusted *p* value < 0.05; Fig. 2c; Supplementary Table 1) and in DiffNPCs, the corresponding number of DEGs was 879 (Fig. 2d; Supplementary Table 1). The number of upregulated DEGs was 734 at the NPC stage and 634 at the DiffNPC stage (Fig. 2e; Supplementary information Table 1). To validate the RNAseq data, we performed quantitative real-time PCR on 10 genes (*HOXB4*, *HOXA3*, *TGFB2*, *EMP1*, *TAGLN2*, *FABP7*, *S100B*, *GFAP*, *VIM*, and *RIBC2*) on RNA derived from four trisomic iPSC lines (two from DS1 and two from DS2, respectively) and three euploid lines (Ctrl1, Ctrl2 and Ctrl9). The results from quantitative real-time PCR correlated well with that from RNAseq (Supplementary Fig. 2) at both the NPCs stage (*R*^2^ = 0.9572, Spearman’s rho, *ρ* = 0.81, *P* = 0.0049) and the DiffNPC state (*R*^2^ = 0.7914, Spearman’s rho, *ρ* = 0.88, *P* = 0.0008).

The findings from transcriptome analysis indicate that T21 results in specific genome wide transcriptional changes, mainly consisting of upregulations. To clarify any additional molecular aberrations in trisomic NPCs and DiffNPCs, respectively, we quantified the relative levels of proteins at both differentiation stages by LC-MS/MS. We created two pairs, including one T21 and one control sample (i.e. DS1/Ctrl1 and DS2/Ctrl2) for proteome analysis. The two pairs were analysed in triplicates at both stages of differentiation. As expected, fewer proteins were detected using LC-MS/MS compared to the number of transcripts, but all detected proteins were matched with transcripts at both differentiation stages. In general, the proteins detected by LC-MS/MS had corresponding mRNAs expressed at relatively high levels. In NPCs, a total of 724 proteins were quantified in both sample pairs from the three replicates of which 132 were differentially expressed proteins (DEPs) when comparing the T21 to control lines (Bonferroni corrected *p* value cut-off = 0.05/724; Fig. 2f; Supplementary Table 2). In DiffNPC, we identified 439 proteins in both sample pairs in all three replicates of which 51 (5.8%) were DEPs (Bonferroni corrected *p* value cut-off = 0.05/439; Fig. 2g; Supplementary Table 2). Out of the 51 DEPs, 27 were upregulated and 24 were downregulated (Fig. 2h). Among the 10 genes quantified by real-time PCR to validate DEGs (Supplementary Fig. 2), TAGLN2, FABP7 and VIM were found to be dysregulated at both the protein and mRNA levels.

Taken together, these findings suggest extensive molecular abnormalities in T21 cells with temporal variations when comparing the two differentiation stages. Furthermore, the abnormalities are detected by both RNA-seq and LC-MS/MS at magnitudes concordant with the sensitivity of each method.

### Temporal Dynamics of HSA21 Genes with Differentiation in Neural Cells with T21

**Fig. 3:**
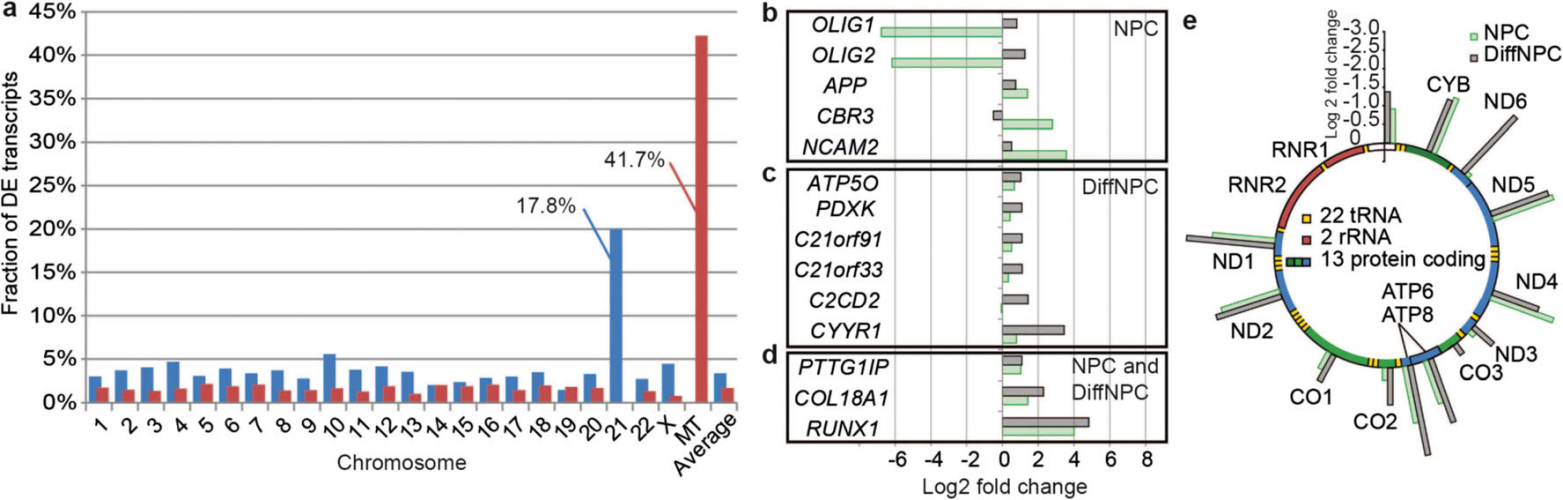
Temporal variation of differential expression from HSA21 genes and the MT genome in T21 lines. **a** Fraction of up- (blue) and downregulated (red) genes per chromosome in DiffNPCs with T21. **b** DEGs on HSA21 that are specific for NPCs with T21 (cut-off *p* < 0.01, absolute log2fold ratio of > 1). **c** DEGs on HSA21 specific for DiffNPCs with T21. **d** DEGs on HSA21 shared at both differentiation stages. **e** Schematic presentation of the MT genome indicating the relative downregulation of MT gene expression (standing bars) in both NPCs (green) and DiffNPCs (grey) with T21

The primary cause of DS is a gene dosage imbalance due to T21, and we then wanted to assess the temporal changes of DEGs on HSA21 at the two differentiation time points. In trisomic NPCs, 10.6% of HSA21 genes were found upregulated compared to an average of 3.4% for non-HSA21 genes (adjusted *p* value < 0.05; Supplementary Table 4). The number of upregulated HSA21 genes in DiffNPCs increased to 17.8% compared to an average of 2.8% on all other autosomes; Fig. 3a; Supplementary Table 3). Using a more stringent approach, we identified altogether 14 DEGs on HSA21 in T21 lines (*p* value < 0.01 and absolute log2 fold change > 1; Fig. 3b–d). The expression levels of many of these genes deviated strongly from an expected 3:2 dosage in the T21 lines. Five of these DEGs were detected exclusively in trisomic NPCs (Fig. 3b). Notably, the transcription factors *OLIG1* and *OLIG2* showed a 6–7-fold downregulation in when compared to euploid NPCs. Given the known effect on myelination processes in DS brain [9], we further investigated the expression of down-stream genes in the myelination process. In DiffNPCs, but not in NPCs, we identified six DEGs (i.e. *EPB41L3*, *HEXB*, *SOD1*, *PMP22*, *NFASC* and *LAMA2*; Supplementary Table 2) that belong to the myelination gene ontology category (GO:0042552) and the general process of ensheathment of neurons (GO:0007272). This confirms an effect on oligodendrocyte differentiation and myelination pathways in our model. In DiffNPCs, we identified six markedly DEGs on HSA21 (*p* value < 0.01; log2 fold change > 1; Fig. 3c). The strongest dysregulations were observed for *CYYR1*, encoding a Shisa-protein implicated in growth factor receptor activity [37], and for *C2CD2*, encoding a transmembrane protein with yet unclear functions. In addition, three HSA21 genes were significantly upregulated in T21 lines at both the NPC and the DiffNPC stages (*p* value < 0.01; log2 fold change > 1; Fig. 3d). The strongest and most consistent upregulation at both differentiation stages was identified for *RUNX1* encoding the transcription factor AML1 (average log2 fold change = 4.4), supporting its recently reported role in DS neurogenesis [38]. Together, these observations indicate that several DEGs on HSA21 implicated in neuronal functions strongly deviate from the predicted 3:2 levels in our trisomic model.

### Mitochondrial Transcription Decreases with Neural Differentiation in T21 Cells

We next asked if the genome wide DEGs in trisomic lines showed any chromosome specific clustering during neurogenesis in addition to that for HSA21. In particular, we observed that 10 out of the 24 expressed mitochondrial (MT) genes (42%; average count of > 5 per transcript in all samples) were significantly downregulated in trisomic DiffNPCs, but not in trisomic NPCs (log2 fold average change 2.6; Fig. 3e). This is in line with previous studies on cardiac tissues and fibroblasts from DS cases supporting a ubiquitous downregulation of mitochondrial activity [39]. At the early NPC stage, the MT genes showed a general but non-significant downregulation (log2 fold average change 1.9). The MT transcripts encoding tRNAs showed a tendency for a reduction with differentiation, albeit from very low expression levels as judged from our euploid lines. To exclude MT gene depletion as a cause of impaired MT transcription, we then analysed the copy number of MT vs. nuclear genes by ddPCR. The relative amount of MT to nuclear genes turned out similar in trisomic and euploid NPCs but the ratio was increased in trisomic DiffNPCs (Supplementary Fig. 4). These observations indicate a pronounced downregulation of MT transcription with differentiation in T21 neural lines despite increase in MT-DNA copy numbers.

### Network Analysis Reveals Dysregulated Pathways and Hub Genes During Neurogenesis in T21 Cells

We then set out to identify dysregulated pathways and altered molecular functions in T21 cells. All differentially expressed transcripts and proteins were used as input data using Enrichr [40]. The analysis revealed a group of highly significant dysregulated pathways (KEGG) in trisomic cells that was shared at both stages of differentiation, e.g. focal adhesion, extracellular matrix (ECM) function and TGF-beta signalling (Enrichr Combined Score > 10; Supplementary Table 4). Similar findings were obtained using gene ontology (GO) enrichment analysis suggesting perturbations in collagen binding and cell matrix adhesion. In addition, several aberrant pathways in trisomic lines were markedly associated with either the NPC or the DiffNPC stage. In trisomic NPCs, but not in DiffNPC, we identified dysregulations of pathways for signalling that regulates pluripotency and synapse formation (KEGG: 04550, 04724 and 04727). This suggests a perturbed transition from the neural stem cell stage as well as a disrupted formation of more mature neuronal structures in trisomic cells [15]. At the DiffNPC stage, another set of perturbed pathways emerged, e.g. glycolysis/gluconeogenesis (KEGG: 00010), axon guidance (KEGG: 04360), long term depression of cerebellum and 04730) and a group of signalling pathways (KEGG: 04151, 04390, 04015, 04933), suggesting that deficits in these functions becomes significant with neural differentiation.

Previous studies have indicated that the progenitor fate choice of glia cells in DS neurogenesis results in a propensity for astrogliogenesis that brings a toxic effect to neurons [19, 41]. Consistent with increased formation of glia cells and astrocytes in DS [42], we observed an elevated expression of the genes, *GFAP*, *VIM* and *S100B* in trisomic lines, mainly at the NPC stage. Increased expression was also observed for the glia cell markers *TAGLN2* and *FABP7* (Supplementary Table 2; Supplementary Table 3; Supplementary Fig. 2). While not as evident as for the dysregulated glia cell markers, we observed a slight reduction in expression of genes for neuronal markers such as *LMX1B* (Supplementary Table 2). To corroborate our findings, we then co-stained DiffNPC with β-III-tubulin, GFAP and DAPI. In euploid lines, 19% of cells stained positive for GFAP, whereas in T21 lines 48% stained positive for GFAP (Supplementary Fig. 3). This supports a marked increase in proportion of glia cells in the DiffNPCs with T21. Together, the molecular profiles suggest dynamic abnormalities of pathways of importance for cell fate, differentiation and energy metabolism in T21 neural cells that correlate with disturbed DS brain development.

We then used Enrichr to identify hub proteins using the protein–protein interaction (PPI) network in our integrated transcriptome and proteome data. Hub proteins are highly connected with other proteins within a given functional module or a pathway that increases the probability for a phenotypic association. In NPCs, we identified 23 hub proteins within significantly altered networks (adj. *p* value < 0.01, *z*-score < 1.1, combined score > 10; Supplementary Table 4). Importantly, transcriptome data indicated that four of these hub proteins were upregulated (adjusted *p* value < 0.05): *VCL* (encoding vinculin), *CAV1* (encoding caveolin-1), *IL7R* (encoding interleukin-7 receptor) and *APP* (encoding amyloid precursor protein). The hub gene *APP* is encoded on HSA21, and the peptides formed from amyloid precursor protein (APP) degradation are the major component of amyloid plaques in Alzheimer’s disease, a frequent complication in DS patients [4]. In addition, APP has a role in promoting growth and plasticity of neural cells [43]. A negative effect of increased APP levels on neurogenesis in T21 complies with the marked upregulation of the *APP* gene at the NPC stage. Furthermore, the increased expression of the hub genes *VCL*, *CAV1* and *IL7R* predicts perturbation of interconnected functions such as focal adhesion, ECM interactions, receptor-mediated signalling and cytoskeletal adaptation. In DiffNPCs, two out of 17 hub genes were found dysregulated (adjusted *p* value < 0.05) and with increased expression: *ITGB1* (encoding Integrin subunit beta-1) and *CAV1*. The membrane bound ITGB1 protein mediates ECM-receptor interaction, focal adhesion, cytoskeletal formation, migration, as well as neural-specific functions such as axon guidance and synapse formation [44]. Furthermore, ITGB1 activates the PI3K-Akt pathway implicated in cell cycle regulation. However, the vast majority of the altogether 40 hub proteins in disturbed functional networks were not dysregulated and suggest a more fine-tuning role in DS neurogenesis (Enrichr combined score > 10). These observations reveal a set of five dysregulated hub genes in perturbed pathways and with dynamic variations along differentiation of trisomic neural lines. These hub genes predict downstream effects on proliferation, maturation, energy metabolism, differentiation and degeneration of cells that comply with abnormalities observed in DS brains and to some extent with trisomic iPSC and orthologous mouse models [4, 21, 45].

### Integrated Transcriptome and Proteome Analysis Disclose Temporal Dynamics of Disturbed Functional Clusters in T21 Neural Cells

**Fig. 4:**
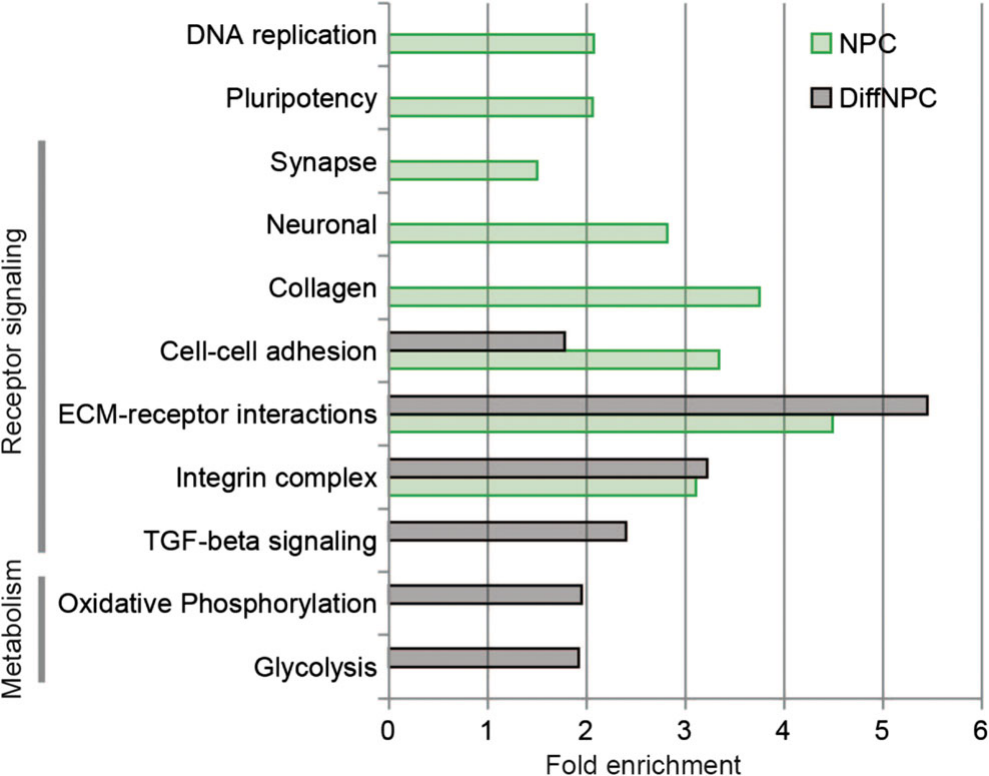
Dynamics of perturbed functional clusters in T21 neural lines based on integrated analysis of data from NPCs and DiffNPCs. Eleven deficient functional clusters were identified based on annotations using DAVID considering all transcriptome and proteome data sets from NPCs and DiffNPCs, respectively. Fold enrichment of clusters was obtained from separate data analysis of NPCs and DiffNPCs, respectively. The clusters were named after representative annotations. Five dysregulated clusters (DNA replication, pluripotency, synapse, neuronal and collagen) were characteristic for trisomic NPCs suggesting a significant impact of these clusters in early T21 neurogenesis. In DiffNPCs, three dysregulated clusters are distinguished (TGF-beta signaling, oxidative phosphorylation and glycolysis) suggesting their ascendant role with differentiation in T21 neurogenesis. Seven dysregulated clusters belong to receptor signaling pathways and these were evident at both stages of differentiation implying a critical role for dysfunctional intercellular communication and ECM in T21. Fold enrichment scores of annotated clusters are shown for NPC (green) and DiffNPC (grey). The complete clustering data are summarized in Supplemental Table S5

**Fig. 5:**
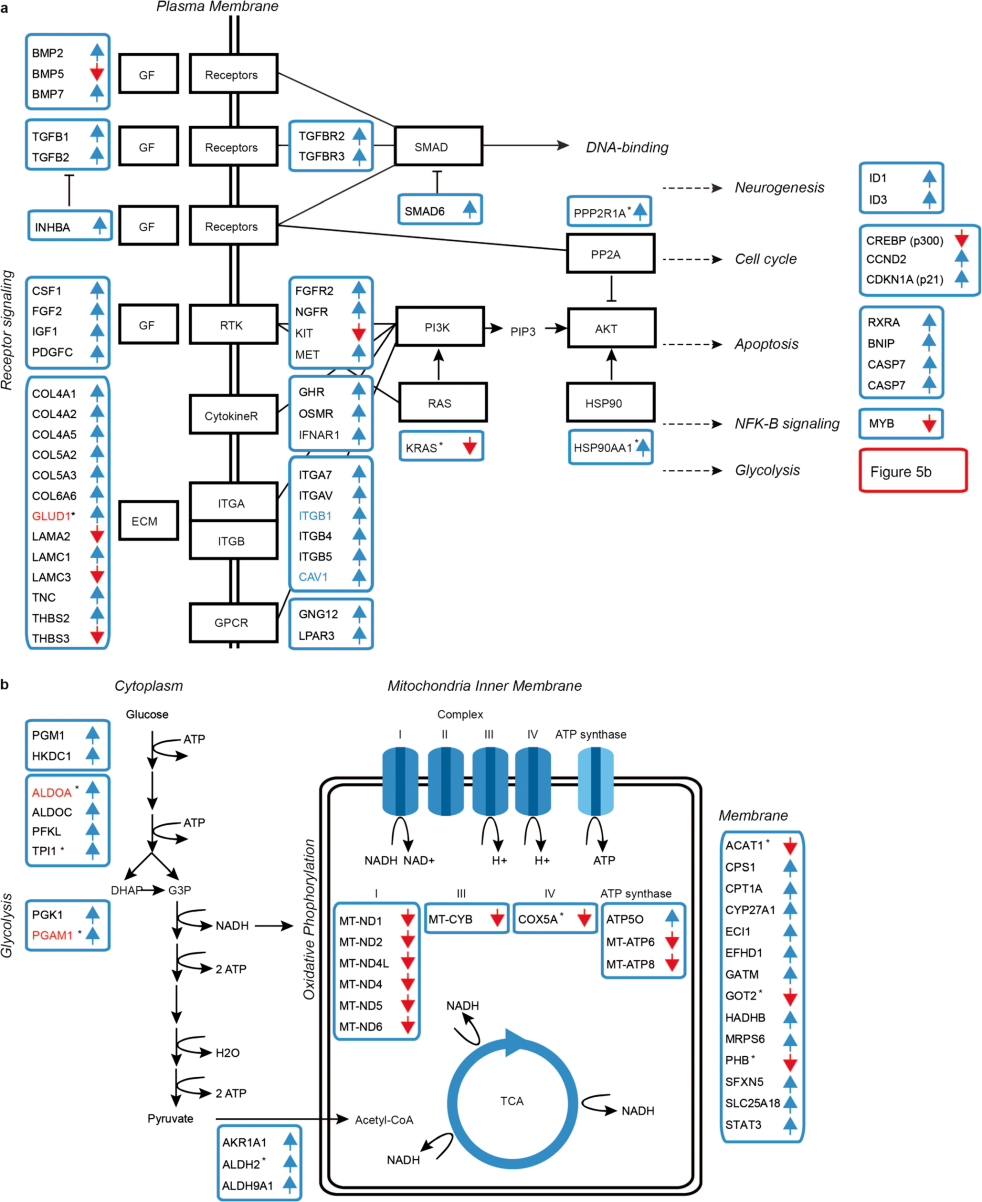
Graphical overview of selected and perturbed functional pathways in DiffNPC with T21. **a** Schematic illustration of DE transcripts and proteins (black abbreviations within blue boxes) integrated in pathways. Elements are indicated as up- (blue arrows) or downregulated (red arrows). DE elements from protein analysis are indicated with a star (asterisk), and DE elements from both transcriptome and proteome analysis are indicated in red letters. Hub genes are indicated in blue letters. Disturbed pathways for collagen, ECM-receptor interactions, integrin complex, receptor tyrosine kinase (RTK) and TGF-beta and growth factor (GF) receptor signaling are shown with their downstream effects on cell cycle regulation, apoptosis, glycolysis and neurogenesis. PM plasma membrane, GF growth factors, ECM extracellular matrix, RTK receptor tyrosine kinases, ITGA/B integrin alpha and beta, GPCR G protein coupled receptors. **b** Highlight from **a** summarizing the molecular abnormalities associated with glycolysis and mitochondrial activity. The functional clusters are annotated using DAVID

To further analyse the dynamics of molecular abnormalities during neural differentiation in T21, we annotated functional clusters using the Database for Annotation, Visualization and Integrated Discovery (DAVID) that takes both pathways and functional modules into account [46]. The analysis, based on both transcriptome and proteome data at either of the two stages of differentiation, revealed 11 unique clusters in trisomic lines (Enrichment score > 1.5; Fig. 4; Supplementary Table 5). Eight of the dysregulated clusters, i.e. DNA-replication, collagen, cell-adhesion, ECM-receptor interactions, integrin complex, TGF-beta signalling, oxidative phosphorylation and glycolysis, agree with findings in fetal heart tissues and fibroblasts from DS cases [10, 39]. This supports paramount and tissue unspecific regulatory abnormalities in DS organogenesis. Five clusters stand out only in trisomic NPCs, i.e. DNA replication, pluripotency, synaptic maturation, neuroactive signalling and collagen binding clusters (Fig. 4). These observations suggest that regulation of cell growth and transition from a progenitor stage are predominant defects at an early stage in trisomic neural cells. Furthermore, the associated perturbation of synapse and neuronal clusters, involving GABAergic, glutamatergic and dopaminergic synapses and signalling, imply early abnormalities in the formation of specific cell progenitors and neural structures. Conversely, three perturbed clusters were apparent only in DiffNPCs, namely TGF-beta signalling, oxidative phosphorylation and glycolysis supporting their ascendant role with neuronal differentiation in T21 cells (Fig. 5a, b). The three deficient clusters are likely consequences from perturbations related to collagen, cell–cell adhesion, ECM-receptor interaction and the integrin complex thus pointing to the fundamental role of disrupted ECM components, cell–cell interactions and cell membrane associated communication in T21 cells along neural differentiation. These deficits presumably reinforce the disturbed metabolic clusters that stand out in DiffNPC. Taken together, the integrated transcriptome and proteome data indicate sequential and dynamic perturbations of major functional clusters during differentiation of trisomic neural cells. Moreover, the temporal dynamics of these major aberrant functions coincide with stages for growth, differentiation and maturation of the developing brain.

Our data correlates well with, to our knowledge, the most extensive meta-analysis based on 45 different DS transcriptome and proteome data sets [47]. This study identified 324 genes showing consistent and significant genome-wide dosage effects associated with T21. Out of the 324 DEGs, 49 (15%) are differentially expressed in our NPCs and 67 (21%) in DiffNPCs. Among the 324 DEGs located on chromosome 21, our study replicated 23% of these genes in NPCs and 38% in DiffNPCs, respectively, including *SOD1*, *APP* and *RUNX1*. Furthermore, we observed that our DEGs are enriched for targets of RUNX1 (Enrichr: ENCODE and ChEA Consensus TFs from ChIP-X; adjusted *p* value 0.042) such as *TGFB2* and *EMP1* at both differentiation stages in line with previous reports (Supplementary Fig. 2) [38, 48].

We then correlated the transcriptional changes associated with our T21 lines to previously data from DS brain regions. When comparing our data with DEGs from the dorsal frontal cortex of DS brains [9], 16 of the 179 DEGs (9%) were replicated in our DiffNPCs. For the chromosome 21 genes, 8 of the 16 DEGs (50%) from dorsal frontal cortex show a higher expression also in our DiffNPC with T21. The numbers are slightly lower for T21 NPCs, i.e. 6% for all chromosomes and only 13% for chromosome 21, respectively, suggesting that the gene expression pattern in trisomic DiffNPC share more abnormalities with the developing DS brain than trisomic NPCs.

### Altered Proliferation, Neurite Formation and ROS Production in Neural Cells with T21

**Fig. 6:**
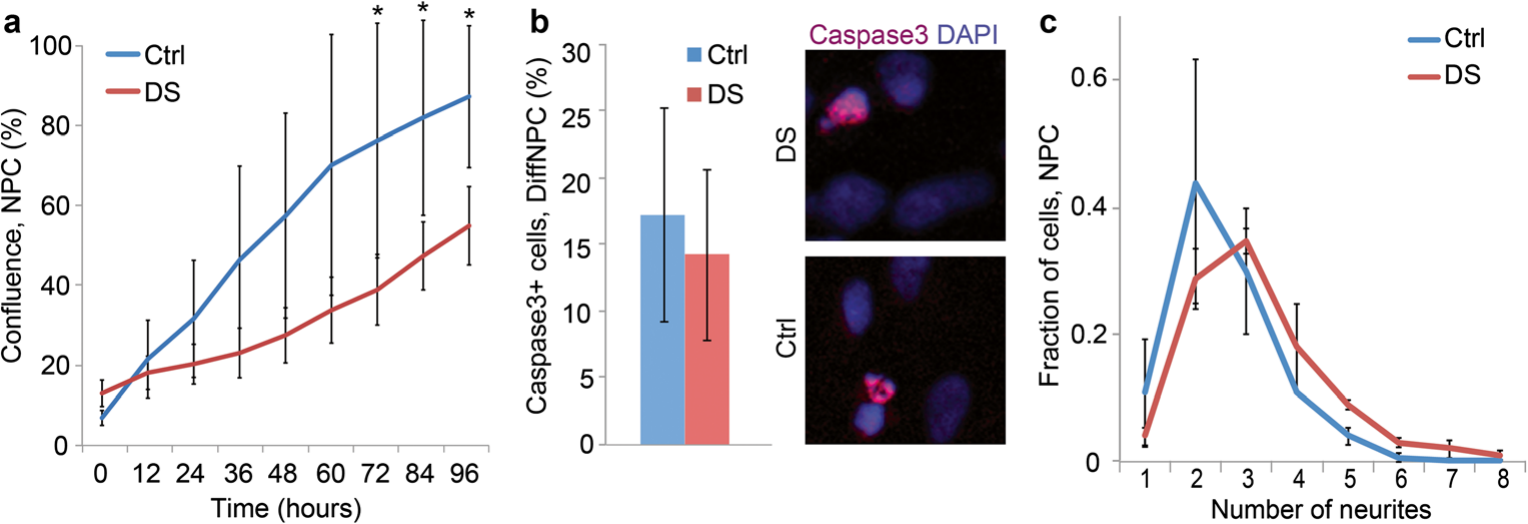
Functional characterization of T21 neural lines. **a** Growth rate of euploid (Ctrl; Ctrl 1 and Ctrl2) and T21 NPCs (DS; DS1 and DS2) for 96 h illustrating reduced proliferation in trisomic lines (mean+/-SD; *: *p* < 0.05). Live-cell imaging with IncuCyte was used for analysis. **b** Proportion of Caspase-3 positive DiffNPCs in T21 (DS) and euploid (Ctrl) lines (left) with representative pictures from both lines (right). No significant difference was detected between the two groups from the analysis of 569 cells with T21 (DS1 and DS2) and 639 euploid cells (Ctrl1 and Ctrl2) (mean+/-SD). Staining was visualized using confocal microscope and ImageJ software. **c** Analysis of neurite outgrowth in β-III-tubulin-positive NPCs. The trisomic NPCs show a different distribution in neurite numbers when compared to euploid NPCs (DS1 and DS2, *N* = 2, *n* = 101, Mo = 3; Ctrl1 and Ctrl2, *N* = 2, *n* = 148, Mo = 2; *F* = 19.375, mean+/-SD, *p* < 0.05)

To assess whether the molecular alterations identified in trisomic cells correlate with cellular abnormalities in our model system, we first examined the growth rate of euploid and trisomic NPCs using a live cell imaging system. The NPC cultures were followed for 96 h, and the T21 neural lines exhibited a markedly reduced proliferation rate (Fig. 6a). Increased apoptosis is unlikely to a cause of the decrease in expansion of cells because staining with caspase-3 revealed similar results in trisomic and euploid lines (Fig. 6b). The reduced cell growth is consistent with the observed and marked dysregulation of elements and pathways in DNA replication, ECM- and growth factor receptor mediated signalling.

Dendritic and synaptic formations are impaired in DS brains [4]. We then investigated β-III-tubulin positive NPCs for the ability to form neurites and to migrate. The trisomic lines showed a different distribution in numbers of neurite outgrowth when compared to euploid cell lines (Fig. 6c). This is in agreement with previous reports on abnormal neurite formation in neural cells with T21 [19] as well as the altered neural connectivity in brains of DS fetuses and orthologous mouse models [45]. However, we were not able to detect any abnormalities in motility of NPCs when tracking displacement and migration distances for 6 h (not shown). In combination, these findings indicate both a reduced proliferative capacity and an altered neurite formation in T21 lines at the NPC stage.

The observed reduction in markers for mitochondrial activity in trisomic DiffNPCs in our study is compatible with an adaptation to cellular stress [49]. The reduction in mitochondrial gene expression accompanied by increased mtDNA copy number (Supplementary Fig. 4) clearly indicates dramatic changes in mitochondrial function. Previous studies have shown that T21 neuronal cells are under stress but with conflicting results regarding production of reactive oxygen species (ROS) [19, 21]. We therefore examined ROS levels under normal culture conditions as well as under hydrogen peroxide (H_2_O_2_) stress in DiffNPCs. Under both conditions, the trisomic cells exhibited ROS levels that were identical to that of euploid neural cells (see Supplementary Fig. 5). Seemingly contradictory, our global gene expression analysis revealed a dysregulation of genes involved in ROS metabolism, antioxidation and apoptosis in both NPCs and DiffNPCs with T21 (Supplementary Table 6). These observations suggest that the DiffNPC with T21 are tolerant to increased cellular stress, possibly due to the large proportion of glia cells using glycolysis or via downregulation of energy metabolism as described previously [49].

## Discussion

The present study was motivated by questions on the temporal dynamics of abnormalities in pathways, elements and functions in DS brain neurogenesis. Recapitulating abnormalities in DS brain using neuronal models with T21 are important for the development of novel therapeutic strategies and to improve cognitive outcome of patients [25]. In this report, leveraging the molecular co-expression network analysis in an iPSC model with T21 during neural differentiation, we provide a framework for a better understanding of the molecular basis of DS brain development. We focused our transcriptome analysis on protein coding sequences at two neural differentiation time points, and the comprehensive molecular analysis, based on data from deep RNA-seq complemented with LC-MS/MS, highlights 11 disturbed functional clusters and their temporal dynamics in T21 neural lines. The interpretation of RNAseq and LC-MS/MS data complies with each other in that they overlap in perturbed elements and molecular pathways. Furthermore, the study highlights the sequential disturbance of major pathways in T21 cells relevant to neuronal developmental processes. For example, the dysregulation of the DNA replication, pluripotency, synaptic formation and neural signalling clusters in NPCs appear as prominent in early stages of neurogenesis when cell proliferation and transition from the stem cell stages are critical [4]. This was accompanied by a reduced proliferative rate and perturbed neurite formation at the NPC stage. The abnormalities in trisomic NPCs related to neuronal ligand–receptor interactions and synaptic formation are consistent with studies of DS brains and T21 models [19, 45]. Furthermore, both trisomic NPCs and DiffNPCs show altered pathways for ECM-receptor interactions, cell–cell communication and integrin complex formation supporting that ECM-related functions and cell membrane–associated communication are disrupted in T21 cells along neural differentiation. Conversely and at the later DiffNPC stage, deficient TGF-beta signalling, SMAD-associated signal transduction and different growth factor receptor activities were evident. Furthermore, glycolysis and oxidative phosphorylation emerged as distinctive dysregulated clusters in trisomic DiffNPCs along with increased downregulation of MT transcription. These perturbed functional clusters observed in trisomic cells are possibly confined to fractions of cells in the mixed population studied and will require further studies. Furthermore, several alterations of functional clusters in our study have previously been demonstrated in other non-neural cell types with T21, e.g. ECM-receptor interactions, cell–cell adhesion, integrin complex and glycolysis, thus indicating general intrinsic defects in DS organogenesis [10, 39]. In addition, many of the molecular and cellular perturbations in our neural lines with T21 agree with an altered cellular composition, impaired trophic interactions and deficient functional neural circuits observed in DS brains [7].

The most dysregulated functional modules revealed altogether 23 genes encoding hub proteins in trisomic NPCs and DiffNPCs. In search for candidate targets for intervention, hub proteins are particularly interesting as they are highly interacting proteins in defined networks and functional pathways. Five out of 23 identified hub genes were markedly upregulated of which *VCL*, *CAV1*, *ITGB1* and *IL7R* encode proteins with inter-connected functions such as focal adhesion and ECM-receptor signalling. These functions are of critical importance for cell-cycle regulation, cell morphology and cytoskeleton plasticity, migration and axon guidance, in agreement with morphological and functional perturbations in DS brains and DS models. Furthermore, the upregulation of the hub gene *APP* on HSA21 agrees with deposition of APP derived peptides and Alzheimer-like neurodegenerative processes occurring early in DS brains [50]. Furthermore, the brain pathology in Alzheimer’s disease has been modelled in iPSCs with T21 [16, 22]. However, emerging evidence indicate that APP is of importance in neurodevelopment by promoting growth and plasticity of neural cells [43]. Our identification of *APP* as an upregulated hub gene at the early NPC stage agrees with these dual roles that may suggest a detrimental role for the protein from early stages of T21 neurogenesis.

Our transcriptome data highlights several DEGs located within the DSCR on HSA21 with marked deviations from the predicted 3:2 expression ratio. Notable examples are downregulation of the transcription factors *OLIG1* and *OLIG2* (Log2 fold change < − 6) in trisomic NPCs but not in DiffNPCs. This coincides with the glia cell fate at an early stage of fetal development, and it is consistent with the deficient myelination observed in DS brains [9]. However, the downregulation contrasts to previously reported over-expression of *OLIG1* and *OLIG2* reported in cells from the ventricular zone of DS fetuses [51] and in iPSC-derived neural cells [15]. These contradicting results may, at least for *OLIG2*, be explained by the differentiation stage and the composition of neuronal cell types analysed [9]. At the later DiffNPC stage, the profile of dysregulated HSA21 genes was different. The most conspicuous example is the *CYYR1* gene (Log2 fold change > 3.5), encoding a highly conserved tyrosine-rich domain. The precise function of the CYYR1 protein is not known, but it has been suggested that the protein has a regulatory function on intracellular growth factor receptor trafficking and thus for receptor mediated signalling [37]. However, the consequence of the strong CYYR1 overexpression on neurogenesis requires further studies. In addition, the HSA21 gene *RUNX1*, encoding the transcription factor AML1, showed upregulations in both NPCs and DiffNPCs with T21 (Log2 fold change > 4). Recently, *RUNX1* was shown to play an important role in neurogenesis and neurite outgrowth [38]. This complies with the altered neurite outgrowth observed in our and previous studies [15] and suggest a direct role for *RUNX1* for disturbed connectivity and synapse formation during DS brain development. Taken together, our observations show that a set of HSA21 genes within the DSCR and with known neural functions are markedly dysregulated at defined differentiation stages in T21 lines supporting direct contributions to abnormal neurogenesis in DS.

The gene dosage imbalance from HSA21 has profound downstream trans-acting effects on the entire genome and, ultimately, on the phenotypic outcome [9, 12]. Our study underscores this tenet and shows that DEGs are distributed on all disomic chromosomes. We further observed a downregulation of the mitochondrial transcriptome in trisomic lines that became marked at the later time point of differentiation in our model. Mitochondrial dysfunction and altered mitochondrial morphology are well-known features in T21 cells [49, 52]. The assessment of molecular perturbations associated with a reduced mitochondrial activity in our trisomic lines is accompanied by an altered stress marker profile from RNAseq and reduced cellular growth. However, this was neither associated with an increase in apoptosis as defined by caspase-3 staining nor with increased ROS levels, despite H_2_O_2_ induction, suggesting that the mixed neural cell populations with T21 are tolerant to increased stress. These findings are consistent with observations in an independent iPSC-derived neural model with T21 [21] but contrast to previous reports on increased ROS levels in trisomic skin fibroblasts and astroglia cells [19] as well as to the increased apoptosis and caspase-3 staining in trisomic NPCs [15]. However, the ROS levels, oxidative stress and cellular function vary with energy requirements of cell types as well as culture conditions that may explain the discrepancy [49]. Furthermore, the impaired MT transcription and increase in transcripts for glycolysis in trisomic cells were accompanied by a reduced proliferative rate in NPCs, a marked increase in the fraction of glia cells and a reduced proportion of neuronal cells. Astroglia cells can store glycogen and have an active glycolysis that can be upregulated, whereas neurons are dependent on the TCA cycle and oxidative phosphorylation for metabolism [53]. The downregulation of mitochondrial transcription in T21 lines may thus, and at least in part, reflect the altered distribution of the two cell types and their different metabolism. This implies that the distinct dysregulation of MT transcription in our mixed T21 neural cultures is downstream of regulatory factors for growth and cell fate decision and not caused by increased ROS levels. The downregulation of mitochondrial transcription in our T21 cultures, and thereby a reduced oxidative phosphorylation, may thus be a consequence of reduced proliferative rate and the cellular composition with different metabolic requirements.

Our high throughput transcriptome and proteome data generated from an iPSC-derived model of DS neurogenesis add several aspects to the previously reported profiles of molecular aberrations due to genomic imbalance of HSA21. The global changes in T21 neural cells identified herein affect an extensive number of pathways that can be assembled in 11 functional clusters with temporal and sequential variations from the analysis of two differentiation stages. We further identify strong dysregulations of a set of key factors associated with neural differentiation in T21 cells. Our data set provides a framework for further studies of candidate targets with the long-term goal to interfere with brain development in DS and for improved cognitive outcome in DS patients.

## Electronic Supplementary Material

ESM 1 (XLS 22 kb)

ESM 2 (XLS 371 kb)

ESM 3 (XLS 56 kb)

ESM 4 (XLS 40 kb)

ESM 5 (XLS 66 kb)

ESM 6 (XLS 27 kb)

ESM 7 (XLS 21 kb)

ESM 8 (DOCX 4061 kb)

ESM 9 (DOCX 19 kb)
