# Supplementary material for: Transcriptome and Proteome Profiling of Neural Induced Pluripotent Stem Cells from Individuals with Down Syndrome Disclose Dynamic Dysregulations of Key Pathways and Cellular Functions
Source: Mol Neurobiol. 2019 Apr 13;56(10):7113–27. doi: 10.1007/s12035-019-1585-3 (PMC6728280; doi:10.1007/s12035-019-1585-3)
Supplement: Supplementary file 8 — (DOCX 4061 kb) [file 12035_2019_1585_MOESM8_ESM.docx]

**Supplementary Figures and legends and legends to supplementary Tables**

**Supplementary Figure 1.**


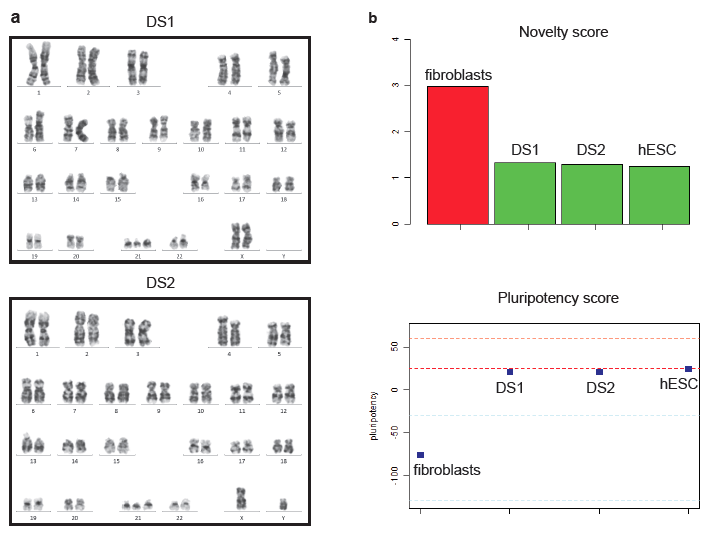


**Genomic integrity and pluripotency of iPSCs with T21. a** Representative karyotypes from G-banded metaphase chromosomes on iPSCs from T21 lines DS1 and DS2. **b** Pluritest analysis of trisomic iPSC lines ([www.pluritest.org](http://www.pluritest.org); [1]) from DS1 and DS2 together with a fibroblast line and a human ESC line [2]. Novelty scores measures the general model matrix inbuilt in PluriTest and reflect the deviation from the expected gene expression pattern in iPSCs. Pluripotency score relates the cell line to two different phenotypic classes: Various types of pluripotent stem cells (between red dashed lines) and somatic/differentiated cells (between blue dashed lines).

**Supplementary Figure 2.**


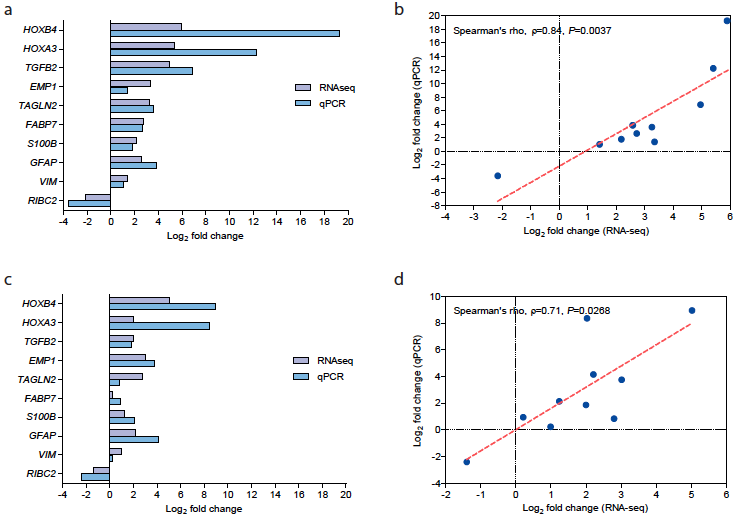


**Quantitative real-time PCR (qRT-PCR) validation of RNAseq data.** Expression were analysed for ten genes showing differential expression from RNAseq and LC-MS/MS in T21 neural lines at either, or both, the NPC and DiffNPC stages. **a** The X-axis shows the differential expression in trisomic lines of RNAseq data (purple) and the expression from qPCR analysis at the NPC stage (blue). Results represent mean values expressed as Log_2_ values of the fold change. **b** Correlation analysis of RNAseq and qPCR data for NPCs (Log2 values of the fold change; Spearman’s rho, ρ=0.84, *P*=0.0037). **c** The X-axis shows the different expression in trisomic lines of RNAseq data (purple) and the expression from qPCR analysis at the DiffNPC stage. Results represent mean values expressed as Log_2_ values of the fold change. **d** Correlation analysis of RNA-seq and qPCR for DiffNPCs (Log2 values of the fold change; Spearman’s rho, ρ=0.71, *P*=0.0268). The qPCR results are based on analysis of mRNA derived from four iPSC clones with T21 (two from DS1 and two from DS2, respectively) and three euploid iPSC clones (Ctrl1, Ctrl2 and Ctrl9). The genes selected for qRT-PCR were dysregulated RNAseq data of NPCs for *HOXB4, HOXA3, TGFB2, EMP1, TAGLN2, FABP7, S100B, VIM, RIBC2*; in RNAseq of DiffNPCs for *HOXB4, HOXA3, TGFB2, EMP1, TAGLN2, VIM*; from LC/MS data of NPCs for TAGLN2 and VIM; and from LC-MS/MS data of DiffNPCs for VIM and FABP7.

**Supplementary Figure 3.**


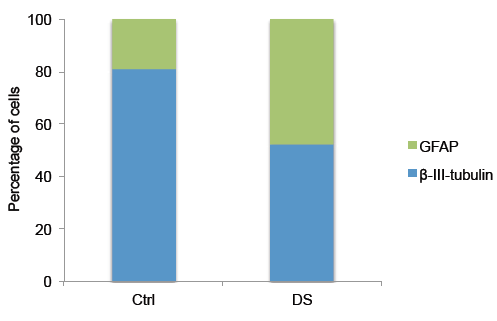


**Increased proportion of GFAP positive cells in DiffNPCs with T21**. Relative distribution of GFAP positive and β-III-tubulin positive DiffNPCs in euploid lines (Ctrl and Ctrl2) and in T21 lines (DS1 and DS2), respectively. Cultures differentiated for 30 days were co-stained with β-III -tubulin, GFAP and DAPI. The euploid lines (Ctrl) contains 81% β-III -tubulin positive cells (n=391) and 19% GFAP positive cells (n=92, 19%). In T21 cultures, 52% of cells were β-III -tubulin positive (n=170) and 48% were GFAP positive (n=154). Cells positive for each marker were counted from three randomly chosen view-fields in each of the four lines. The results are consistent with the previously described "glialisation" of neural cell cultures with T21.

**Supplementary figure 4.**


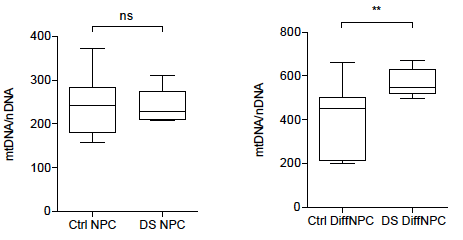


**Mitochondrial to nuclear DNA copy number ratio increases with differentiation in T21 neural cells.** Ratio between the mtDNA copy number and the nuclear copy number in euploid and T21 neural cells at the NPC stage (left) and the DiffNPC (right) stages. The analysis is based on probes targeting the mitochondrial *ND1* gene and the nuclear *HBB* gene. The copy number ratio did not differ between trisomic and euploid lines at the NPC stage (left). In DiffNPCs, the copy number of mtDNA to nDNA was significantly increased in T21 lines when compared to euploid lines (**: P<0.01, Student’s two-sided t-test; ns: non-significant).

**Supplementary Figure 5.**


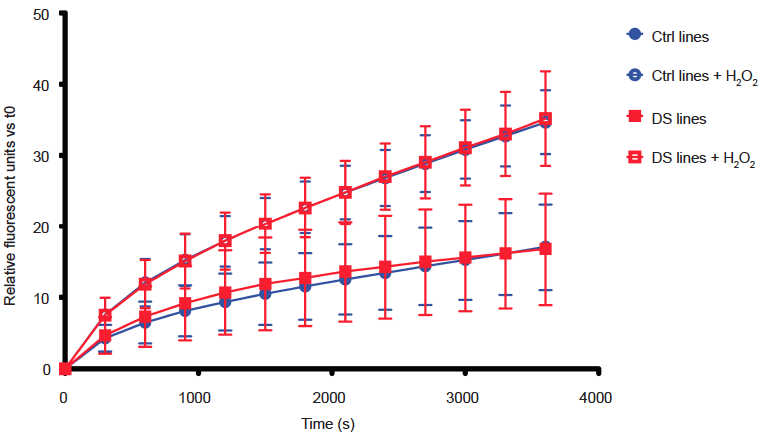


**Intracellular reactive oxygen species (ROS) are not increased in DiffNPCs with T21**. Graph illustrating increase of intracellular ROS in trisomic (DS1 and DS2, n=6; red squares) and euploid (Ctrl1 and Ctlr2, n=8; blue circles) DiffNPCs with (open symbols) and without (filled symbols) stress induction using hydrogen peroxide (H2O2). No differences in ROS levels were detected between euploid and trisomic lines.

**Legends to supplementary Tables:**

**Supplementary Table 1.** Key genes used for clustering analysis of NPCs, DiffNPCs and Brainspan samples. Protocol specific differentially expressed genes, i.e. genes expressed exclusively in induced pluripotent stem cells (iPSC), NPC and DiffNPC, respectively, derived from two euploid cell lines (Ctrl 1 and Ctrl2).

**Supplementary Table 2 (two tabs).** Differentially expressed genes (DEGs) in trisomic NPC (Table 1.1) and DiffNPC (Table 1.2) based on adjusted p-value (p<0.05) and sorted by log2 fold change.

**Supplementary Table 3 (two tabs).** Differentially expressed proteins (DEPs) in trisomic NPCs (Table 2.1) and DiffNPC (Table 2.2) based on Bonferroni corrected p-value (p<0.05) and sorted by log2 fold change.

**Supplementary Table 4 (two tabs).** Differentially expressed genes (DEGs) on HSA21 and from MT genome in trisomic NPCs (Table 3.1) and DiffNPC (Table 3.2) based on adjusted p-value (p<0.05) and sorted by relative chromosomal position. Righ-tmost columns indicates the number and fraction of genome-wide up- or down-regulated transcripts per chromosome.

**Supplementary Table 5 (two tabs).** Perturbed KEGG pathways, GO molecular functions, chromosomal location and PPI hub proteins in trisomic NPCs (Table 4.1) and DiffNPCs (Table 4.2) from annotational clustering using EnrichR. Functional annotations from KEGG pathways, GO molecular function, chromosomal location and PPI hub proteins that reached Benjamini corrected p-value of <0.05 and a Combined score >10 derived from EnrichR. PPI hub proteins that are differentially expressed from the transcriptome analysis is annotated with * and from the proteome analysis with # (all are upregulated in T21).

**Supplementary Table 6 (two tabs).** Perturbed biological functions in trisomic NPCs (Table 5.1) and DiffNPCs (Table 5.2) from annotational Clustering using DAVID. Functional clusters are based on integrated analysis of both transcriptomes and proteomes in NPCs and DiffNPCs, where at least one annotation category reached Benjamini corrected p-value of <0.05.

**Supplementary Table 7.** Differential expression of stress related markers. The “DEG column” indicates whether the gene is differentially expressed in either NPC or DiffNPC, or at both differentiation stages. Genes are sorted by chromosomal location.

**References**

1. Muller FJ, Schuldt BM, Williams R, Mason D, Altun G, Papapetrou EP, Danner S, Goldmann JE, Herbst A, Schmidt NO, Aldenhoff JB, Laurent LC, Loring JF (2011) A bioinformatic assay for pluripotency in human cells. Nat Methods 8 (4):315-317. doi:10.1038/nmeth.1580

2. Lappalainen RS, Salomaki M, Yla-Outinen L, Heikkila TJ, Hyttinen JA, Pihlajamaki H, Suuronen R, Skottman H, Narkilahti S (2010) Similarly derived and cultured hESC lines show variation in their developmental potential towards neuronal cells in long-term culture. Regenerative medicine 5 (5):749-762. doi:10.2217/rme.10.58
