# Supplementary material for: Transcriptome and Proteome Profiling of Neural Induced Pluripotent Stem Cells from Individuals with Down Syndrome Disclose Dynamic Dysregulations of Key Pathways and Cellular Functions
Source: Mol Neurobiol. 2019 Apr 13;56(10):7113–27. doi: 10.1007/s12035-019-1585-3 (PMC6728280; doi:10.1007/s12035-019-1585-3)
Supplement: Supplementary file 9 — (DOCX 19 kb) [file 12035_2019_1585_MOESM9_ESM.docx]

**Supplementary Materials and Methods**

**RNA sequencing and transcriptome analysis**

To make the published Brainspan data of human brain transcriptomes comparable to our gene counts, Brainspan RPKM values were transformed by substituting each expression value x with log2(x+1), while the read counts for our own data were transformed using voom() in the R limma package. In addition, the ComBat function in the R sva package was used to attempt to adjust for study-specific effects. In ComBat, only the study (“Brainspan” or “own”) was used for the correction. The log-transformed, combat-corrected data visualized using the Rtsne implementation of t-distributed stochastic neighbourhood embedding (t-SNE).

For the identification of differentially expressed transcripts, a Benjamini-Hochberg adjusted p-value (p-adjusted) cut off of 0.05 was considered significant. To assess overall similarity between samples we calculated the Euclidean distance between samples using transformed (rlog) counts from DESeq2 and dist() illustrated using the pheatmap package in R.

The web-based annotation tool Enrichr was used for functional annotations of DE gene and implements three approaches to compute enrichment. The first one is the Fisher exact test and the second test is z-score on a correction to the Fisher exact test based on intuition. Lastly a combined score is calculated based on the p-value and the z-score of the deviation from the expected rank.

Statistical analysis using DAVD (<https://david.ncifcrf.gov>) was performed on functional enrichment using Benjamini corrected p-values. Markers for neuronal cell differentiation were taken from Abcams neural markers guide (Abcam).

**Mass-spectrometry and proteome analysis**

Briefly, the samples of cell pellets were homogenized in an SDS-containing buffer. The total protein concentration in the samples was measured using the DC Protein Assay with bovine serum albumin (BSA) as standard. Aliquots corresponding to 15 μg protein were taken out from each sample, precipitated using acetone and re-suspended in a urea containing buffer and in solution digested using trypsin. The resulting peptides were purified on Stage Tips and labelled using dimethyl labelling, separated in reversed-phase on a C18-column, using a 240 min long gradient, and electrosprayed on-line to a Q Exactive Plus Orbitrap mass spectrometer (Thermo Finnigan). Tandem mass spectrometry was performed applying HCD. Protein identification and quantitation was performed using the quantitation software MaxQuant 1.5.1.2. The three RAW data files from each comparison were combined into one search respectively in the software. The database for protein identification contains human proteins extracted from the Swissprot database (Release April 2015). A decoy search database, including common contaminants and a reverse database was used to estimate the false discovery rate (FDR). The search criterion for protein identification and quantification was set to at least two identified peptides of 99% confidence level per protein. Protein ratios (Ratio H/L) that where different from 1 between patients and control cells where identified using triplicate measurements of ratios by a one-sample t-test against a null hypothesis of ratio = 1 (no differential expression). The resulting p-values where combined using Fisher's combined probability test. The results where Bonferroni corrected for the number of identified proteins.

A decoy search database, including common contaminants and a reverse database was used to estimate the false discovery rate (FDR). The search criterion for protein identification and quantification was set to at least two identified peptides of 99% confidence level per protein. Protein ratios (Ratio H/L) that where different from 1 between patients and control cells where identified using triplicate measurements of ratios by a one-sample t-test against a null hypothesis of ratio = 1 (no differential expression). The resulting p-values where combined using Fisher's combined probability test. The results where Bonferroni corrected for the number of identified proteins.

**Validation of RNA-sequencing data and quantification of mitochondrial DNA**

Altered expression levels of 10 selected genes was confirmed by quantitative real-time PCR (q-RTPCR) utilizing the same samples as for RNAseq. We further added mRNA from two independent lines derived from DS1 and DS2, respectively, as well as from one control line (Ctrl9). Primers were designed with the Primer-BLAST online tool. High-Capacity cDNA Reverse Transcription Kit (Applied Biosystems) was used for cDNA synthesis added to FastStart Universal SYBR Green Master (Rox) mix (Sigma-Aldrich) according to manufacturer’s protocol. StepOnePlus™ Real-Time PCR System (Applied Biosystems) and StepOne Software v2.2.2 were applied for analysis. The genes selected for qRT-PCR were dysregulated by RNAseq in NPCs for *HOXB4, HOXA3, TGFB2, EMP1, TAGLN2, FABP7, S100B, VIM, RIBC2*; in DiffNPCs for *HOXB4, HOXA3, TGFB2, EMP1, TAGLN2, VIM*. The genes selected for qRT-PCR were dysregulated by LC/MS in NPCs for TAGLN2, VIM; and in DiffNPCs for VIM and FABP7.

The expression levels of the genes were measured using the following primers:

*HOXB4* F: 5'-CTGGATGCGCAAAGTTCAC-3', R: 5'-AGCGGTTGTAGTGAAATTCCTT-3';

*HOXA*3 F: 5'-GCAGCTCCAGCTCAGGCGAA-3', R: 5'-GCCGGCACAGGTAGCGGTTG-3';

*TGFB2* F: 5'-ATTGATGGCACCTCCACATAT-3', R: 5'-ACGTAGGCAGCAATTATCCTG-3';

*EMP1* F: 5'-CCCTCCTGGTCTTCGTGT-3', R: 5'-GGAATAGCCGTGGTGATA-3'; *TAGLN2* F: 5'-GGACGCGAGAACTTCCAG-3', R: 5'-TTCCCAGAGGTCCACAGTTT-3';

*FABP7* F: 5'-ACAGAAATGGGATGGCAAAG-3', R: 5'-CTCATAGTGGCGAACAGCAA-3';

*S100B* F: 5'-AGGGAGACAAGCACAAGCT-3', R: 5'-CCGTCTCCATCATTGTCCA-3';

*GFAP* F: 5'-AAAGAGATCCGCACGCAGTA-3', R: 5'-AGGTCAAGGACTGCAACTGG-3';

*VIM* F: 5'- GCAGGAGGAGATGCTTCAGA-3', R: 5'- TCGTGGAGTTTCTTCAAAAAGG-3';

*RIBC2* F: 5'-CCGAGATCACCAACCTCCT-3', R: 5'-ACTGCTGCCGCTCAAACAG-3';

*ACTB* F: 5'-CAGGAGGAGCAATGATCTTGATCT3', R: 5'-TCATGAAGTGTGACGTGGACATC-3'.

Mitochondrial DNA (mtDNA) and nuclear (nDNA) copy number was determined using ddPCR system and an automated droplet generator and reader (QX200 Droplet Digital PCR, Bio-Rad). Reactions containing ddPCR™ Supermix for Probes (no dUTP), HEX and FAM-labeled probes and 1ng of template DNA were fractionated into ~20,000 droplets according to manufacturer’s instructions. For mtDNA quantification, we used a probe targeting *MT-ND1* encoding NADH dehydrogenase 1 and for nDNA quantification we used a probe for the *HBB* gene [1] obtained from Bio-Rad. The ratio of mtDNA/nDNA was calculated for statistics using Student’s two-sided t-test.

**References**

1. Wachsmuth M, Hubner A, Li M, Madea B, Stoneking M (2016) Age-Related and Heteroplasmy-Related Variation in Human mtDNA Copy Number. PLoS Genet 12 (3):e1005939. doi:10.1371/journal.pgen.1005939
